# Optimization of Bio-Based Polyurethane Elastic Nanofibrous Membrane via Electrospinning for Waterproof and Breathable Applications

**DOI:** 10.3390/polym17040486

**Published:** 2025-02-13

**Authors:** Bin Zhang, Xueqin Li, Yanyan Lin, Ningbo Cheng, Wenling Jiao, Xianfeng Wang, Jianyong Yu, Bin Ding

**Affiliations:** 1 Shanghai Frontier Science Research Center of Advanced Textiles, College of Textiles, Donghua University, Shanghai 201620, China; 2 Innovation Center for Textile Science and Technology, Donghua University, Shanghai 201620, China

**Keywords:** bio-based polyurethane, waterproof, breathable, electrospinning, nanofibrous membranes

## Abstract

Bio-based polyurethane (BPU) offers excellent biocompatibility and outstanding elasticity, providing vast potential for the development of next-generation waterproof and breathable materials. However, achieving stable and uniform electrospinning of BPU remains a significant challenge. Herein, BPU with superior electrospinning performance was synthesized using poly(butylene sebacate), poly(trimethylene ether glycol), ethylene glycol, and methylene diphenyl diisocyanate (MDI) as raw materials. BPU nanofibrous membranes were successfully fabricated using solutions of varying concentrations (12 wt%, 16 wt%, 20 wt%, and 24 wt%), and their morphology, mechanical properties, hydrophobicity, and breathability were systematically analyzed. The nanofibrous membrane prepared with 20 wt% BPU solution exhibited optimal fiber morphology and mechanical properties, with a tensile strength of 15.6 MPa and an elongation at break of 440.8%. In contrast, lower concentrations (12 wt% and 16 wt%) resulted in insufficient fiber formation, leading to poorer performance, while higher concentrations (24 wt%) significantly reduced fiber uniformity, negatively impacting the overall performance. Additionally, the nanofibrous membrane produced from the 20 wt% BPU solution demonstrated significant hydrophobicity and breathability, with a water contact angle of 133.2°, hydrostatic pressure of 48.2 kPa, and breathability of 12.6 kg·m^2^·d^−1^. These findings suggest that BPU nanofibrous membranes produced via electrospinning hold great potential for application in functional textiles.

## 1. Introduction

Waterproof breathable membranes (WBMs) represent a significant advancement in material science, combining excellent water repellency with high water vapor permeability [1,2,3]. This unique combination of properties results in exceptional wearer comfort and is the focus of extensive research across various fields, including outdoor apparel, smart textiles, and medical protective equipment [4,5,6]. The traditional materials used to manufacture WBMs include polyacrylonitrile [7], polyvinylidene fluoride [8], and polytetrafluoroethylene (PTFE) [9]. However, these traditional materials frequently exhibit several limitations, including limited flexibility, poor biocompatibility, environmental concerns, and a lack of renewability [10]. As awareness of sustainability increases and environmental regulations become more stringent, the demand for bio-based materials is steadily rising, driven by their environmental benefits [11,12]. Bio-based polyurethane (BPU) represents a new class of environmentally friendly materials that are partially or fully derived from renewable resources [13,14,15]. BPUs not only offer the same excellent mechanical properties, flexibility, and water resistance as traditional petroleum-based polyurethanes, but also provide significant advantages in terms of environmental impact, health, and safety [16,17]. Consequently, BPUs are emerging as promising alternatives to conventional PUs and have already demonstrated considerable potential for applications in fields such as biomedicine and functional textiles [18,19,20].

Electrospinning is an advanced nanofiber fabrication technique that produces fibrous membranes with high porosity, fine fiber diameters, and small internal pores, making it a promising method for preparing WBMs [21,22,23,24]. Nevertheless, polyurethane (PU) poses a significant challenge for consistent and stable electrospinning, primarily due to its chemical diversity and relatively high viscosity [25,26]. Researchers are exploring novel techniques and methodologies to improve the spinnability of PU solutions. For instance, modifying the molar ratio of polyol to isocyanate [27], optimizing the PU solution formulation [28], and incorporating functional additives [29,30,31] are effective strategies to enhance the spinnability and fiber structure of PU solutions. Gradinaru et al. [32] developed a magnetic nanocomposite fiber mat by incorporating iron oxide (Fe_3_O_4_) nanoparticles into a PU solution. The inclusion of Fe_3_O_4_ nanoparticles enhanced the electrical conductivity of the spinning solution, resulting in a reduction in the diameter of the electrospinning PU fibers. Banikazemi et al. [28] optimized the electrospinning performance of PU by adjusting the solvent type and found that a 60:40 ratio of DMF to chloroform was the optimal solvent combination. They also observed that the fiber morphology was excellent at a solution concentration of 5 *w*/*v*%. While various methods have been developed to enhance the electrospinning performance of PU nanofibrous membranes, the diversity in BPU synthesis still poses a challenge for the rapid and stable production of BPU nanofibers with optimal morphology and properties. In our preliminary study [33], we demonstrated that BPU, with polyester and polyether polyols as soft chain segments, can significantly improve flexibility while maintaining a certain level of strength. This enhancement is beneficial for achieving electrospinning of BPU and improving the flexibility of BPU nanofibrous membranes. Therefore, the careful selection of appropriate BPU raw materials and spinning conditions is an effective approach to overcoming the technical challenges associated with BPU electrospinning.

In this work, we demonstrated BPU nanofibrous membranes that can be rapidly and stably produced by electrospinning, which exhibit significant waterproof and breathable properties. Poly(butyl sebacate) (PBSe), poly(trimethylene ether glycol) (PO3G), ethylene glycol (EG), and diphenylmethane diisocyanate (MDI) were employed in the synthesis of PBSe/PO3G-BPU. Compared with traditional petroleum-based polyurethanes, PBSe/PO3G-BPU not only exhibits significant mechanical properties but also offers renewability and a lower environmental footprint. Furthermore, this study systematically explored how different concentrations of PBSe/PO3G-BPU solutions influence the morphology and properties of nanofibrous membranes. The optimal results were observed at a solution concentration of 20 wt%, where nanofibers exhibited the best balance between structure and performance, enabling the controlled fabrication of waterproof and breathable nanofibrous membranes. These results present a promising approach for developing electrospinning BPU nanofibrous membranes with good waterproof and breathable characteristics.

## 2. Materials and Methods

### 2.1. Materials

PO3G (M_w_ = 2000) was purchased from SK Chemicals Co., Ltd. (Seongnam-si, Republic of Korea). PBSe (M_w_ = 1800) was obtained from Huide Science & Technology Co., LTD. (Shanghai, China). The MDI and EG were purchased from TCI Development Co., Ltd. (Tokyo, Japan). The following materials were purchased from Aladdin Chemicals Co., Ltd. (Los Angeles, CA, USA): dibutyltin dilaurate (DBTDL), N,N-dimethylformamide (DMF), trichloromethane (CHCl_3_), and tetrahydrofuran (THF). The polyethylene film utilized in the comparative skin sensitivity test was purchased from Vinniv Household Products (Taizhou, China). All materials were received in their original state.

### 2.2. Synthesis of PBSe/PO3G-BPU

**Figure 1:**
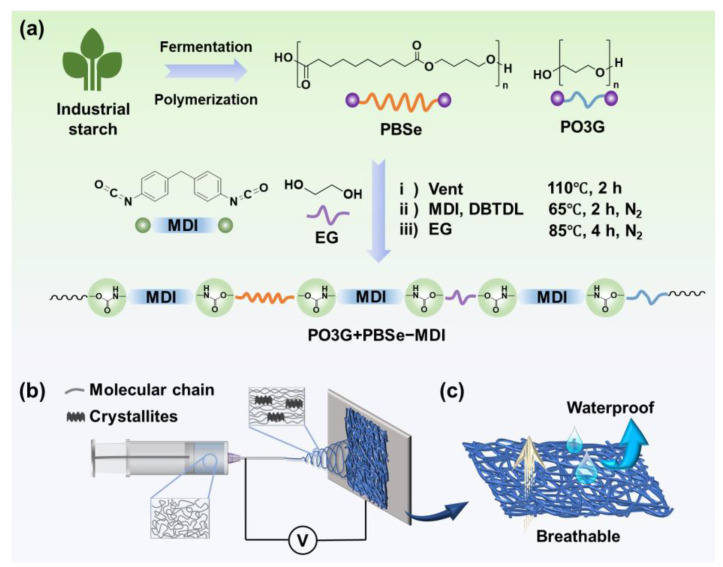
(**a**) Synthesis route of PBSe/PO3G-BPU from PBSe, PO3G, MDI, and EG. Schematic illustration of (**b**) the preparation PBSe/PO3G-BPU nanofibrous membranes and (**c**) the mechanism of PBSe/PO3G-BPU nanofibrous membranes.

The synthesis route of PBSe/PO3G-BPU is illustrated in Figure 1a. Initially, the PBSe and PO3G were combined in a flask and subjected to vacuum drying at 110 °C for a period of 2 h, with the objective of eliminating residual water. Subsequently, the mixture was cooled to 65 °C, and MDI, an appropriate quantity of DMF, and a few drops of DBTDL were added and stirred for two hours to prepare the prepolymer. The prepolymer was subsequently subjected to a four-hour reaction at 85 °C with EG, resulting in the synthesis of PBSe/PO3G-BPU. The molar ratios of PBSe, PO3G, EG, and MDI were 1:1:2:4, ensuring a 1:1 OH-group-to-NCO-group ratio. Once the reaction was complete, the polymer solution was heated to 120 °C for a period of 10 h, allowing for the sufficient removal of the DMF and the production of a pure PBSe/PO3G-BPU solution.

### 2.3. Preparation of PBSe/PO3G-BPU Nanofibrous Membranes

The one-step preparation of PBSe/PO3G-BPU nanofibrous membranes via electrospinning is illustrated in Figure 1b. The PBSe/PO3G-BPU was dissolved in DMF in order to prepare the precursor electrospinning solutions, with concentrations of 12 wt%, 16 wt%, 20 wt%, and 24 wt%. The electrospinning process was conducted in a controlled laboratory setting with 55 ± 5% relative humidity and at 23 ± 2 °C. The electrospinning parameters were set as follows: 15 cm (needle-to-collector distance), 28 kV (needle-to-collector voltage), 1 mL·h^−1^ (injection rate), and 50 r·min^−1^ (collector rotational speed). Under these conditions, PBSe/PO3G-BPU nanofibrous membranes were successfully prepared.

### 2.4. Characterizations

The chemical structure of PBSe/PO3G-BPU dissolved in CHCl_3_ was ascertained through nuclear magnetic resonance (NMR) analysis, employing a full digital superconducting nuclear magnetic resonance spectrometer (Avance III HD 600MHz, Bruker, Bremen, Germany). Fourier transform infrared (FTIR) spectra were obtained using a Nicolet IS10 FTIR spectrometer (Thermo Fisher Scientific, Waltham, MA, USA) for the purpose of analyzing the functional groups present on the surface of the substance. The molecular weight and molecular weight distribution of PBSe/PO3G-BPU dissolved in THF were determined by gel permeation chromatography (GPC) using a PL-GPC220 (Agilent, Santa Clara, CA, USA). X-ray diffraction (XRD) spectra were collected using a D8 advance (Bruker, Germany) to analyze the crystallinity of the samples. The thermal and crystalline behavior of PBSe/PO3G-BPU was studied by differential scanning calorimetry (DSC) using a DSC250 (TA, Newcastle, DE Westlake, OH, USA).

The morphology of the BPU nanofibrous membranes was examined using field emission scanning electron microscopy (FE-SEM, SU5000, Hitachi, Tokyo, Japan). The viscosity of the BPU solution was measured using an LVDV-1T digital rotational viscometer (Fangrui, Shanghai, China). The mechanical properties of the nanofibrous membranes were evaluated using a universal testing machine (35TM-10, Instron, High Wycombe, UK). Specifically, the membranes were cut into 10 mm wide strips and subjected to a tensile test at a rate of 10 mm·min^−1^ until failure, and then tested for cyclic performance at a strain rate of 50%·min^−1^.

Figure 1c illustrates the waterproof and breathable mechanisms. The membrane displays hydrophobic characteristics, with water droplets exhibiting difficulty in penetrating the fibers directly. Meanwhile, the gaps between the fibers permit the passage of water vapor, thereby conferring the material with both waterproof and breathable properties. Subsequent characterization was conducted to further examine the hydrophobicity and breathability of the material. The WVT rate was determined under conditions of 50% relative humidity and 38 °C using a YG601H (Ningfang, Ningbo Xingtai, China) in accordance with ASTM E96 standards [34]. The water contact angle with a 2 μL water droplet was determined using an SL200B goniometer (Kino, Boston, MA, USA). Hydrostatic pressure measurements were conducted using a YG812C (Hongda, Nantong Shenzhen, China) at a rate of increase in water pressure of 6 kPa per minute.

## 3. Results and Discussion

### 3.1. Chemical Structure of PBSe/PO3G-BPU

**Figure 2:**
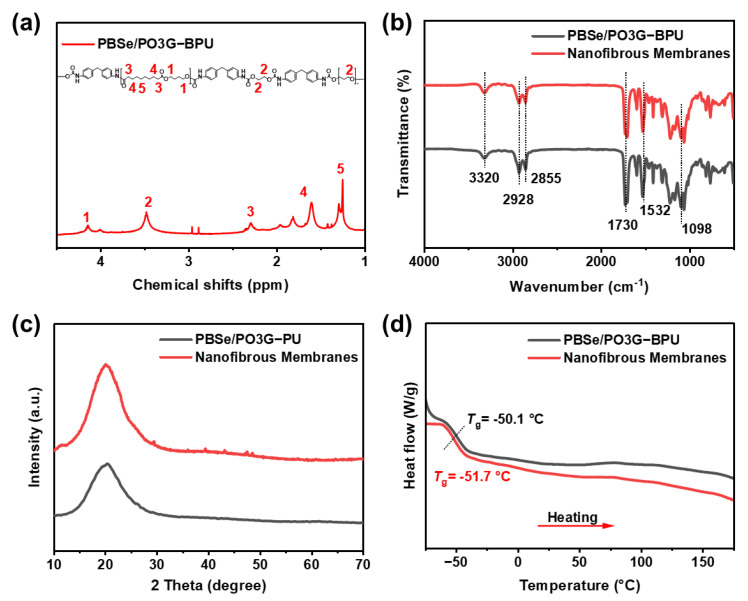
(**a**) The chemical structure of PBSe/PO3G-BPU and its corresponding ^1^H NMR. (**b**) FTIR spectra, (**c**) XRD patterns, (**d**) DSC heating curve of PBSe/PO3G-BPU and nanofibrous membrane prepared from PBSe/PO3G-BPU.

The ^1^H NMR spectrum of PBSe/PO3G-BPU was used to determine its chemical structure, as shown in Figure 2a. The chemical shifts indicated in the spectrum correspond to hydrogen atoms located at specific positions within the molecular structure. The chemical shift at 4.14 ppm is attributed to the methylene hydrogen (–CH_2_–) adjacent to the ether bond (–O–) in PBSe [35,36]. The chemical shift around 3.48 ppm (peak 2) is assigned to the methylene hydrogen (–CH_2_–) adjacent to the ether bond (–O–) in EG and PO3G [37]. The peak near 2.3 ppm (peak 3) is typically associated with the methylene group situated adjacent to the ester group. Consequently, it can be classified as the methylene group directly linked to the one connected to the carbonyl group in PBSe [17]. Peaks between 1.61 and 1.82 ppm (peak 4) correspond to methylene groups adjacent to the carbonyl group, exhibiting a slightly higher chemical shift than the alkyl chain. This shift is attributed to the ortho-methylene groups in the PBSe ester linkage. Peaks between 1.25 and 1.29 ppm (peak 5) represent typical signals for long-chain aliphatic methylene groups (–CH_2_–), primarily originating from the methylene segments of PBSe. These results preliminarily confirm the successful integration of bio-based polyols PBSe and PO3G into the BPU structure as soft chain segments.

To further validate the successful preparation of PBSe/PO3G-BPU, FTIR analysis was conducted, the results of which are presented in Figure 2b. The absorption bands at 3320 cm^−1^ and 1532 cm^−1^ were attributed to the stretching vibration and deformation vibration of the (–NH–) group, respectively, which indicated the presence of urethane bonds in the BPU species. The peaks at 2928 cm^−1^ and 2855 cm^−1^ correspond to the asymmetric and symmetric stretching vibrations of the –CH_2_– group, respectively [38]. The carbonyl (C=O) stretching bands in urethane and PBSe were observed near 1730 cm^−1^. The stretching vibration of the ether bond (C–O–C) is reflected by the absorption peak around 1098 cm^−1^, confirming the presence of soft segments. Meanwhile, the characteristic absorption of the isocyanate group (–NCO) was not detected at 2270 cm^−1^, indicating that the reaction was complete and that no residual isocyanate or solvent was present in the polyurethane [39,40]. As shown in Figure 2b, the surface groups of the PBSe/PO3G-BPU nanofibrous membranes were consistent with those of the PBSe/PO3G-BPU, indicating that the chemical structure of the PBSe/PO3G-BPU was effectively preserved during nanofiber formation. The physicochemical properties of the fibers were uniform throughout the sample, demonstrating excellent controllability.

Furthermore, the molecular weight and molecular weight distribution of the PBSe/PO3G-BPU samples were determined using GPC. As shown in Figure S1, the GPC curve of PBSe/PO3G-BPU exhibited a narrow profile, indicating effective control over the polymerization reaction. The polydispersity index of the synthesized PBSe/PO3G-BPU was 1.37, suggesting a relatively uniform molecular weight distribution. The weight average molecular weight was determined to be 201,151, while the number average molecular weight was approximately 146 kDa. The narrow molecular weight distribution and higher molecular weight impart good spinnability to the PBSe/PO3G-BPU.

The amorphous structure of the PBSe/PO3G-BPU elastomer films was confirmed by XRD spectroscopy (Figure 2c), as no distinct crystalline peaks were observed. In contrast, in addition to the diffraction peak at 20° representing the soft segments [41,42], the PBSe/PO3G-BPU nanofibrous membrane also exhibited a broad diffraction peak around 42° in the XRD pattern, which partially indicates an increase in the crystallinity of the hard segments. During the electrospinning process, the PBSe/PO3G-BPU underwent stretching, which promoted molecular alignment, increased intermolecular interactions between the hard chain segments, and enhanced crystallinity. DSC analysis was performed to further investigate the thermal properties of PBSe/PO3G-BPU (Figure 2d). The glass transition temperature (T_g_) of the soft chain segments in the PBSe/PO3G-BPU nanofibrous membranes was determined to be −51.7 °C, slightly lower than the T_g_ of −50.1 °C observed for PBSe/PO3G-BPU elastomers. This decrease in T_g_ suggests that the stretching and molecular orientation during electrospinning may have relaxed the amorphous structure of the soft chain segments, thereby improving the flexibility and ductility of the fibrous membranes. No T_g_ corresponding to the hard segments was observed in either sample, likely due to the strong hydrogen bonding or van der Waals forces between the MDI-based hard chain segments, which resulted in a more stable molecular structure, preventing any significant glass transition behavior.

These results indicate that electrospinning induces molecular chain rearrangement and microphase separation in PBSe/PO3G-BPU, resulting in structural changes in both the hard and soft segments. Specifically, the crystallinity of the hard segments is enhanced, which improves the material’s structural stability and mechanical properties. Meanwhile, the T_g_ of the soft segments decreases, enhancing the material’s flexibility and extensibility. Therefore, the changes in the microphase separation structure of PBSe/PO3G-BPU during electrospinning contribute to achieving a balance between high rigidity and excellent flexibility.

### 3.2. Microstructure of PBSe/PO3G-BPU Nanofibrous Membranes

**Figure 3:**
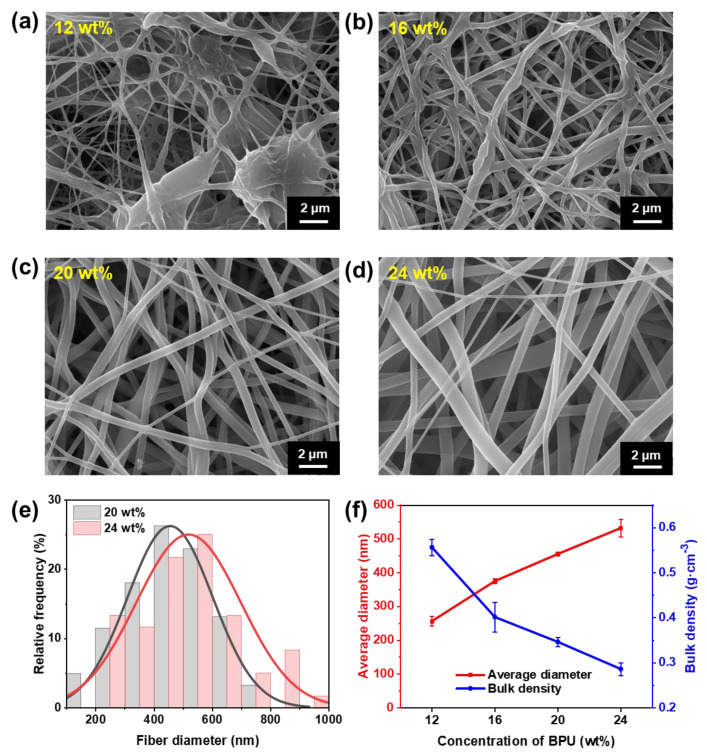
(**a**–**d**) FE-SEM pictures of nanofibrous membranes with varying PBSe/PO3G-BPU concentrations. (**e**) Fiber diameter distribution of nanofibrous membranes prepared from 20 wt%, 24 wt% PBSe/PO3G-BPU solution. (**f**) Statistical graph of average fiber diameter and bulk density of the PBSe/PO3G-BPU nanofibrous membranes.

Figure 3a–d illustrate SEM images of PBSe/PO3G-BPU nanofibrous membranes. It is evident that the solution concentration has a direct impact on the stretching ability of the polymer chains and the fiber formation process. Additionally, the fiber morphology initially improves and then deteriorates with an increase in concentration. This phenomenon is frequently associated with the entanglement of polymer macromolecular chains in solution [43,44,45,46], which is reflected by the rapid increase in viscosity of the PBSe/PO3G-BPU solution as the concentration rises (Figure S2). At a solution concentration of 12 wt%, the formation of beaded structures on the fiber surface was evident. This phenomenon may be attributed to the relatively weak interaction force between the polymer chains at lower concentrations, which resulted in insufficient stretching of the fibers during the electrospinning process. Consequently, discontinuous beaded chains were formed. Upon increasing the solution concentration to 16 wt%, the PBSe/PO3G-BPU was observed to form fibers, yet notable thickened regions and intermolecular adhesion persisted. This may be attributed to the augmented forces between the polymer chains at this concentration, yet the elevated viscosity renders it inadequate for the formation of uniformly drawn fibers. Furthermore, a higher solvent percentage permits a slower evaporation rate, which facilitates the formation of fiber adhesion during the process of fiber formation. At a solution concentration of 20 wt%, the formation of fibers is optimal, and the resulting fiber morphology is relatively uniform. At this concentration, the viscosity of the solution is moderate, providing sufficient stretching force during electrospinning while maintaining good flowability, thus promoting fiber continuity and uniformity. A comparison of the fiber diameters and distributions (Figure 3e) reveals that the fiber-forming effect is diminished at concentrations exceeding 24 wt%. This is accompanied by an increase in fiber diameter and a reduction in fiber uniformity, which can be attributed to the poor fluidity of the solution at higher concentrations. The lack of sufficient stretching of the polymer chains at these concentrations results in greater resistance during fiber formation, which in turn affects the uniformity of the fibers. This indicates that at elevated concentrations, the poor fluidity of the solution may result in augmented resistance during fiber formation, impeding the complete extension of the polymer chains and consequently influencing the homogeneity of the fibers, leading to an increase in fiber diameter and a greater degree of heterogeneity. Therefore, fibers at a concentration of 20 wt% exhibited enhanced morphological and structural stability, rendering them more appropriate for subsequent application studies.

The statistical analysis of fiber diameter and bulk density for nanofibrous membranes formed at different concentrations, using the same spinning duration, is presented in Figure 3f. The results show that as the concentration of the spinning solution increases, the fiber diameter also increases, while the bulk density of the fiber membrane decreases. This suggests that higher solution concentrations lead to increased viscosity, which slows the stretching rate of the solution and reduces the efficiency of fiber formation. As a result, fewer fibers are deposited per unit volume, leading to an increase in membrane porosity. The observed trends of increasing fiber diameter and decreasing membrane weight reflect the combined effects of solution concentration on both the stretching behavior during fiber formation and the fiber deposition within the membrane. These findings underscore the crucial role of concentration in regulating structural formation during the spinning process.

### 3.3. Properties of PBSe/PO3G-BPU Nanofibrous Membranes

**Figure 4:**
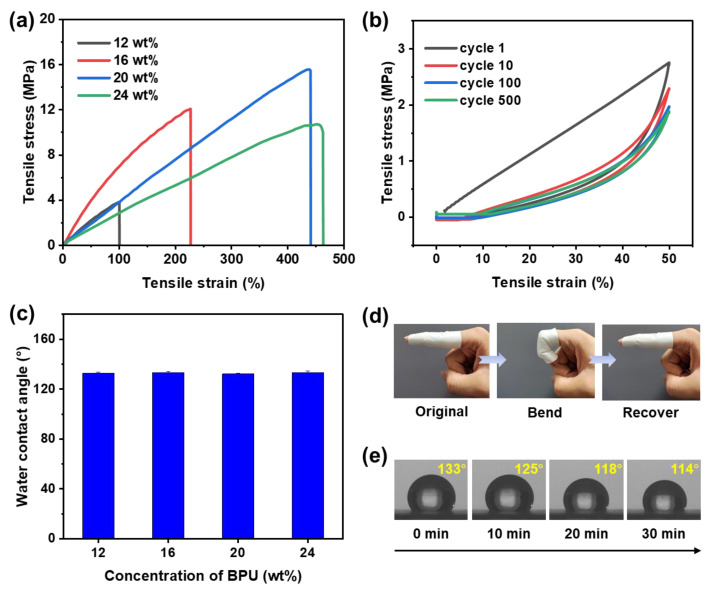
(**a**) Tensile properties of the PBSe/PO3G-BPU nanofibrous membranes. (**b**) A 500-cycle tensile fatigue test with 50% strain of the nanofibrous membranes prepared from 20 wt% PBSe/PO3G-BPU solution. (**c**) Water contact angle of different PBSe/PO3G-BPU nanofibrous membranes. Photographs illustrating (**d**) the elasticity and (**e**) dynamic measurements of water droplet permeation of the nanofibrous membranes prepared from 20 wt% PBSe/PO3G-BPU solution.

The mechanical properties of nanofibrous membranes are of paramount importance with regard to their practical applications. Figure 4a depicts the stress–strain behavior of various PBSe/PO3G-BPU nanofibrous membranes subjected to tensile testing. The stress–strain curves indicate that the behavior of PBSe/PO3G-BPU is analogous to that of a typical elastomer, which also suggests that its hard chain segments are dispersed within the soft chain segments [47]. Table S1 provides a summary of the elongation at break, tensile strength, and Young’s modulus of these membranes. When the spinning solution concentration was 12 wt%, the nanofibrous membrane exhibited the lowest stress and strain. This phenomenon can be attributed to the low viscosity of the spinning solution at lower concentrations, which results in insufficient fiber stretching. As a result, more polymer is used to form beads rather than nanofibers, affecting the structural integrity of the membrane and leading to reduced mechanical strength and ductility. Upon increasing the concentration to 16 wt%, a notable enhancement in the nanofibrous membrane’s strength was observed, indicating that the enhanced fiber morphology contributed to a substantial improvement in the membrane’s overall strength. At this juncture, the Young’s modulus reached its maximum value, which may be attributed to the fiber adhesion phenomenon, resulting in enhanced stiffness. Upon increasing the concentration to 20 wt%, the nanofibrous membranes exhibited optimal mechanical properties, displaying tensile strength of 15.6 MPa and elongation at break of 440.8%. At this concentration, the BPU solution approached the ideal spinning viscosity, producing fibers with moderate thickness, uniform diameters, and a well-defined surface structure. This resulted in enhanced membrane strength and stability. However, at a concentration of 24 wt%, a decrease in tensile strength was observed concomitant with a slight increase in elongation at break. This phenomenon may be due to the excessive concentration leading to uneven or overly coarse fiber formation. Such an irregular fiber structure resulted in varying mechanical properties across different regions of the membrane, making it susceptible to weak spots, which ultimately affected the overall structural stability and strength of the membrane. In conclusion, the concentration of the spinning solution exerted a significant influence on the mechanical properties of the PBSe/PO3G-BPU nanofibrous membranes. The PBSe/PO3G-BPU prepared at 20% concentration offers an optimal balance between strength and flexibility, rendering it suitable for further application studies.

The 500-cycle loading–unloading curves of 20 wt% PBSe/PO3G-BPU nanofibrous membranes (Figure 4b) demonstrated a linear loading curve and a nonlinear unloading curve in the initial cycle, resulting in the formation of hysteresis return lines. In subsequent cycles, both the loading and unloading curves became nonlinear, and the hysteresis loop area gradually decreased. This phenomenon is typically associated with the Mullins effect [48,49]. During the initial loading phase, substantial stress relaxation and microstructural damage occurred within the material’s internal structure. This could manifest as the slippage of chain segments or the rupture of interactions within the packing network. This resulted in linear behavior during the loading phase, while the unloading phase exhibited nonlinearity. As the number of cycles increased, the internal structure of the material became increasingly stable, leading to a reduction in energy dissipation. Consequently, the area of the hysteresis loop (i.e., the energy lost) diminished gradually, and both the loading and unloading curves approached more nonlinear behavior. Concurrently, the Young’s modulus of the subsequent cycles was markedly diminished (Figure S2) and stabilized in comparison to the initial Young’s modulus of the first cycle, indicating that the membrane became softer and provided a more comfortable sensation. Therefore, in certain long-term wear scenarios, this allows the material to better conform over time, thus maintaining comfort during wear. The dynamic bending process of the nanofibrous membrane was simulated by wrapping the membrane around a finger (Figure 4d). The results demonstrate that the finger can bend with minimal effort and that the nanofibrous membrane is capable of not only recovering its shape in response to finger movement but also maintaining a secure fit. This further substantiates the excellent elasticity and comfortable wearability of the PBSe/PO3G-BPU nanofibrous membrane.

The electrospinning process does not result in any alteration to the chemical structure of the polymer. Consequently, the nanofibrous membranes prepared with varying concentrations of spinning solutions exhibited comparable water contact angles (Figure 4c). Furthermore, when the contact time of the water droplet was extended to 30 min, the water contact angle of the nanofibrous membrane samples prepared from a 20% PBSe/PO3G-BPU solution exhibited only a slight decrease (Figure 4e), which provided additional evidence that they demonstrated excellent water resistance.

**Figure 5:**
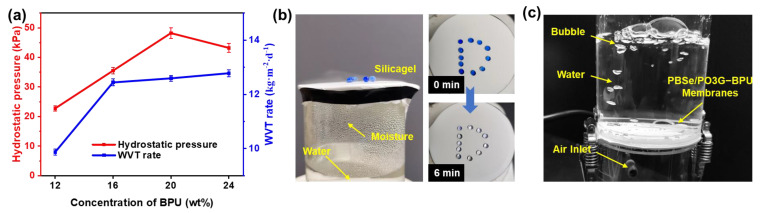
(**a**) Hydrostatic pressure, WVT rate of the PBSe/PO3G-BPU nanofibrous membranes. Photographs illustrating (**b**) water vapor permeability and (**c**) waterproof and breathable properties.

To further validate the potential application of PBSe/PO3G-BPU nanofibrous membranes in waterproof and breathable materials, we conducted a series of experiments to measure the hydrostatic pressure and water vapor transmission rate (Figure 5a). As the concentration of the nanofiber precursor solution increased, the fiber structure and porosity of the membranes were optimized, resulting in an increase in both the hydrostatic pressure and the WVT rate. At a concentration of 20 wt%, the properties approached a maximum, exhibiting hydrostatic pressure of 48.2 kPa and breathability of 12.6 kg·m^2^·d^−1^. Table S2 highlights the performance of PU WBMs from various studies, indicating that the PBSe/PO3G-BPU nanofibrous membrane exhibits excellent overall performance. Notably, it demonstrates superior elongation at break, tensile modulus, and WVT rate compared to other membranes. This excellent strength, extensibility, and breathability contribute to a better wearing experience when in close contact with the skin.

To provide a visual demonstration of the breathability of the nanofibrous membrane, the membrane was placed on a beaker filled with hot water with silica gel on top (Figure 5b). It was evident that the membrane permitted the passage of water vapor, and after a period of six minutes, the silica gel exhibited a discoloration, thereby indicating that the nanofibrous membrane was permeable. This excellent breathability allows for the rapid evaporation of moisture during high temperatures, effectively dissipating body heat and preventing heat discomfort or skin irritation [50]. Figure 5c illustrates the nanofibrous membrane’s dual functionality, exhibiting both waterproof and breathable properties. This exceptional combination of waterproofing and breathability effectively blocks external contaminants while allowing excess moisture to escape, ensuring comfort and dryness even under harsh weather conditions. These results highlight the significant potential of PBSe/PO3G-BPU nanofibrous membranes as highly effective waterproof and breathable materials.

## 4. Conclusions

In this study, a BPU waterproof and breathable nanofibrous membrane was successfully fabricated through a one-step electrospinning process. This BPU, using PBSe and PO3G as soft segments, combines the advantages of polyester and polyether polyurethanes, offering excellent spinnability. As a result, compared to traditional membranes, this nanofibrous membrane not only overcomes the issue of insufficient flexibility found in conventional WBMs but also simplifies the electrospinning process. A comprehensive analysis of the membrane’s morphology, mechanical properties, hydrophobicity, and breathability was conducted. The study revealed that the electrospinning process significantly enhanced the crystallinity of the PBSe/PO3G-BPU hard segments while increasing the molecular mobility of the soft segments. These characteristics endowed the fibers with exceptional flexibility and improved mechanical performance. Additionally, at a BPU solution concentration of 20%, the resulting nanofibrous membrane exhibited tensile strength of 15.6 MPa and elongation at break of 440.8%, demonstrating outstanding flexibility that enhances its durability during wear. The water contact angle was 133.2°, the hydrostatic pressure reached 48.2 kPa, and the breathability was 12.6 kg·m^2^·d^−1^, indicating excellent waterproof and breathable properties that ensure dryness and comfort during wear. The efficient method proposed in this study for the rapid construction of bio-based waterproof and breathable nanofibrous membranes shows great potential. These materials can be extended to other fields, such as biomedical devices and architectural membrane materials, demonstrating broad application value. This research is expected to drive the advancement of bio-based polyurethane materials and the development of waterproof and breathable membrane technologies.

## Data Availability

The original contributions presented in this study are included in the article and Supplementary Material. Further inquiries can be directed to the corresponding authors.

## Supplementary Materials

The following supporting information can be downloaded at https://www.mdpi.com/article/10.3390/polym17040486/s1: Figure S1: GPC traces of PBSe/PO3G-BPU. Eluent: THF; Figure S2: The viscosity of different concentrations of PBSe/PO3G-BPU solutions; Figure S3: Young’s modulus of 500 cyclic tensile fatigue test with 50% strain of 20 wt% PBSe/PO3G-BPU nanofibrous membrane; Table S1. Summary of mechanical properties of PBSe/PO3G-BPU nanofibrous membranes with different concentrations; Table S2. Summary of PU-based WBMS performance in different works. References [51,52] are cited in the supplementary materials.
