# Supplementary material for: Optimization of Bio-Based Polyurethane Elastic Nanofibrous Membrane via Electrospinning for Waterproof and Breathable Applications
Source: Polymers (Basel). 2025 Feb 13;17(4):486. doi: 10.3390/polym17040486 (PMC11859813; doi:10.3390/polym17040486)
Supplement: Supplementary file 1 [file polymers-17-00486-s001.zip › polymers-3403181-supplementary.pdf]

Supplementary Materials

Optimization of bio-based polyurethane elastic nanofibrous membrane via electrospinning for waterproof and breathable applications

Bin Zhang[1,+], Xueqin Li[1,+], Yanyan Lin[1,2] , Ningbo Cheng[1], Wenling Jiao[2,*], Xianfeng Wang[1,2,*], Jianyong Yu[2] and Bin Ding[2]

1 Shanghai Frontier Science Research Center of Advanced Textiles, College of Textiles, Donghua University, Shanghai 201620, China

2 Innovation Center for Textile Science and Technology, Donghua University, Shanghai 201620, China

* Correspondence: wxf@dhu.edu.cn (X.W.); wenlingjiao@dhu.edu.cn (W.J.)

\+ These authors contributed equally to this work

The supporting information contains:

Figures S1−S3
Table S1-S2

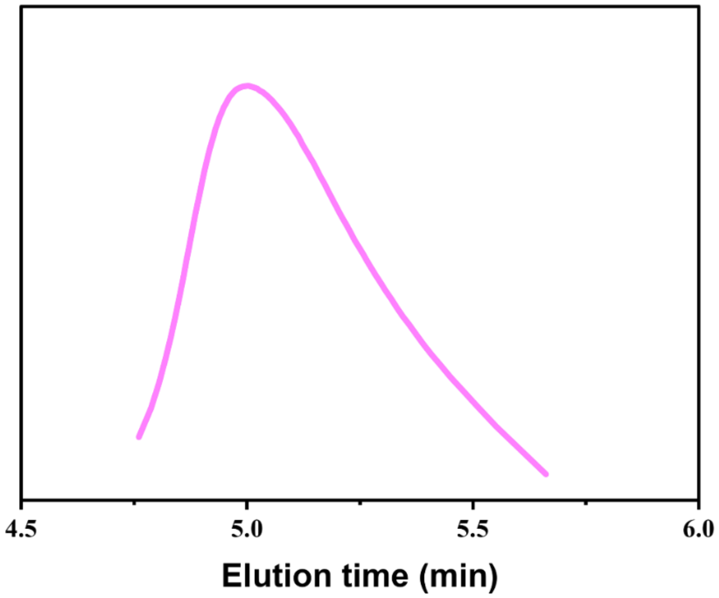

Figure S1. GPC traces of PBSe/PO3G-BPU. Eluent: THF

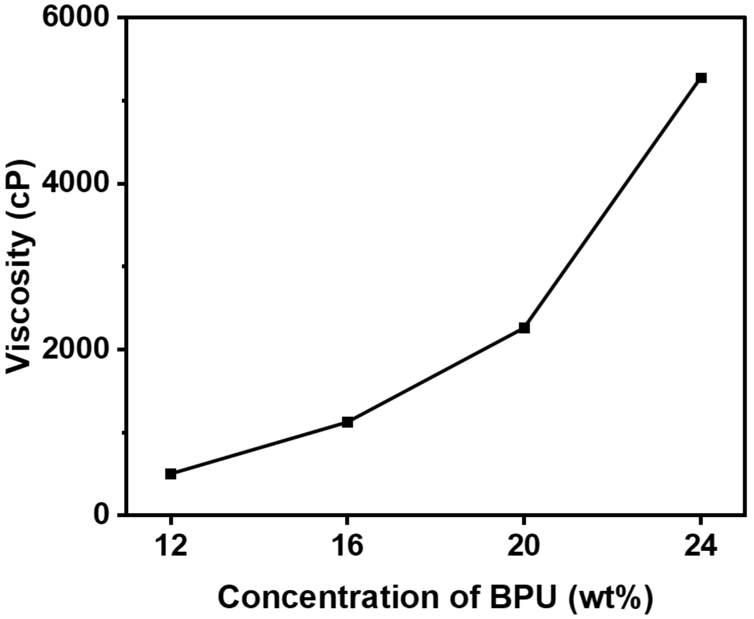

Figure S2. The Viscosity of different concentrations of PBSe/PO3G-BPU solutions.

Table S1. Summary of mechanical properties of PBSe/PO3G-BPU nanofibrous membranes with different concentrations.

Concentration of PBSe/PO3G-BPU (wt%)	Elongation at break (%)	Tensile strength (MPa)	Young's modulus (MPa)
12	100.8	3.9	5.0
16	227.4	12.1	8.5
20	440.8	15.6	3.9
24	463.3	10.3	2.6

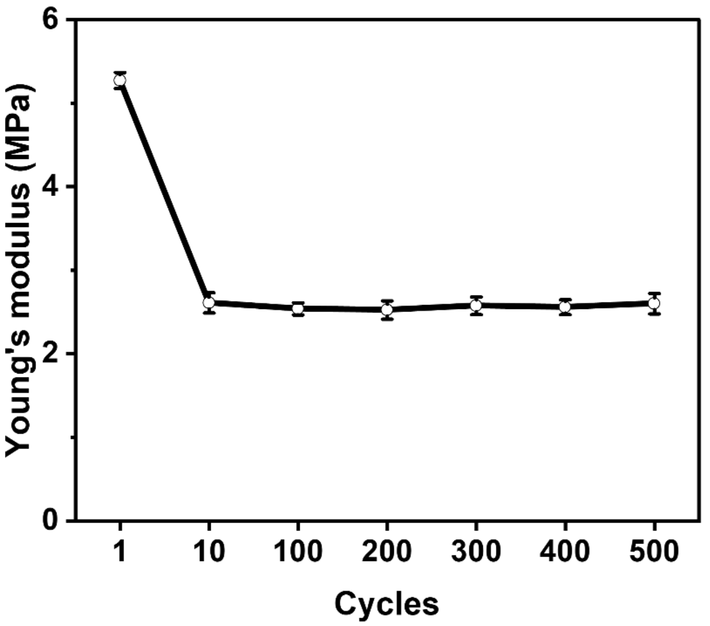

Figure S3. Young's modulus of 500 cyclic tensile fatigue test with a 50% strain of 20 wt% PBSe/PO3G-BPU nanofibrous membranes.

Table S2. Summary of PU-based WBMS performance in different works.

Work	Elongation at break (%)	Tensile strength (MPa)	WVT rate ($kg{\cdot}m^{2}{\cdot}d^{-1}$)	Hydrostatic pressure (KPa)
**This work**	**440.80**	**15.62**	**12.59**	**48.2**
Ref 1[1]	124	10.4	9.06	126.1
Ref 2[2]	265.5	14.1	9.50	54.1
Ref 3[3]	310	-	10.40	50
Ref 4[4]	257	15.3	6.30	93.4
Ref 5[5]	118.11	6.60	8.20	52.3

References

1. Gu, J.; Gu, H.; Cao, J.; Chen, S.; Li, N.; Xiong, J. Robust Hydrophobic Polyurethane Fibrous Membranes with Tunable Porous Structure for Waterproof and Breathable Application. Appl. Surf. Sci. 2018, 439, 589–597, doi:10.1016/j.apsusc.**2017**.12.267.
2. Zhu, W.; Zhao, J.; Wang, X.; Liu, X.; Yu, J.; Ding, B. Facile Fabrication of Fluorine-Free Breathable Poly(Methylhydrosiloxane)/Polyurethane Fibrous Membranes with Enhanced Water-Resistant Capability. J. Colloid Interface Sci. **2019**, 556, 541–548, doi:10.1016/j.jcis.2019.08.092.
3. Yu, Y.; Liu, Y.; Zhang, F.; Jin, S.; Xiao, Y.; Xin, B.; Zheng, Y. Preparation of Waterproof and Breathable Polyurethane Fiber Membrane Modified by Fluorosilane-Modified Silica. Fibers Polym. **2020**, 21, 954–964, doi:10.1007/s12221-020-9562-z.
4. Li, Y.; Zhang, X.; Si, Y.; Yu, J.; Ding, B. Super-Elastic Fluorinated Polyurethane Nanofibrous Membranes with Simultaneously Waterproof and Breathable Performance. ACS Appl. Polym. Mater. **2022**, 4, 5557–5565, doi:10.1021/acsapm.2c00622.
5. Yu, Y.; Xu, G.; Zhao, P.; Zhang, J. Biocompatible, Robust, Waterproof and Breathable PDMS-Based PU Fibrous Membranes for Potential Application in Wound Dressing. Mater. Today Commun. **2024**, 38, 107870, doi:10.1016/j.mtcomm.2023.107870.
